# Bilateral Adrenal Hemorrhage Following Femoral Hip Hemiarthroplasty: A Case Report

**DOI:** 10.7759/cureus.27748

**Published:** 2022-08-07

**Authors:** Athanasios Patousis, Panagiotis Patousis, Georgios Barbakis, Nikolaos P Sachinis

**Affiliations:** 1 First Orthopaedic Department, Aristotle University of Thessaloniki, Thessaloniki, GRC; 2 Department of Orthopaedics and Trauma, General Hospital "Georgios Papanikolaou", Thessaloniki, GRC; 3 Department of Medicine, University Hospital of Ioannina, Ioannina, GRC; 4 Department of Orthopaedics and Trauma, General Hospital of Florina, Florina, GRC; 5 School of Physical Education and Sport Science, National and Kapodistrian University of Athens, Athens, GRC

**Keywords:** fracture femoral neck, massive pulmonary embolism, deep vein thrombosis (dvt), heparin induced thrombocytopenia (hit), bilateral adrenal haemorrhage, partial hip arthroplasty

## Abstract

Bilateral adrenal hemorrhage (BAH) is a rare and potentially fatal complication following total hip arthroplasty and low-molecule heparin use for DVT-prophylaxis. We present a case of a 64-year-old woman who sustained a femoral neck fracture, which was addressed with hip hemiarthroplasty. Twelve days postoperatively DVT was diagnosed and therapeutic doses of low-molecule-heparin were administered. The next day, CTPA was done searching for pulmonary embolism but BAH was shown and a short synacthen test confirmed the diagnosis of adrenal insufficiency. A therapeutic protocol with hydrocortisone was followed.

## Introduction

Heparin and anticoagulation therapy following hip arthroplasties have rarely been the cause of adrenal hemorrhage. Bilateral adrenal hemorrhage (BAH) is an extremely rare condition with an increased fatality incidence. Adrenal insufficiency results from hemorrhage and prompts catastrophic clinical outcomes and low survival rates [1-3]. Misdiagnosis of the syndrome may occur. The main risk factors which induce adrenal insufficiency include heparin-induced thrombopenia (HIT) syndrome, hypercoagulation, thrombocytopenia, stress, hypertension, patient postoperative condition, and sepsis [3]. According to current knowledge, several cases have been reported of heparin-induced thrombocytopenia causing BAH after total hip arthroplasty [4-6]. However, to our knowledge, no case so far has been presented associating BAH and hip hemiarthroplasty, combined with a therapeutic dose of low-molecule heparin due to DVT. This presentation follows a 64-year-old woman who has sustained a femoral neck fracture, which was addressed with hip hemiarthroplasty, as well as the diagnosis, management, and treatment of BAH complications postoperatively.

## Case presentation

**Figure 1:**
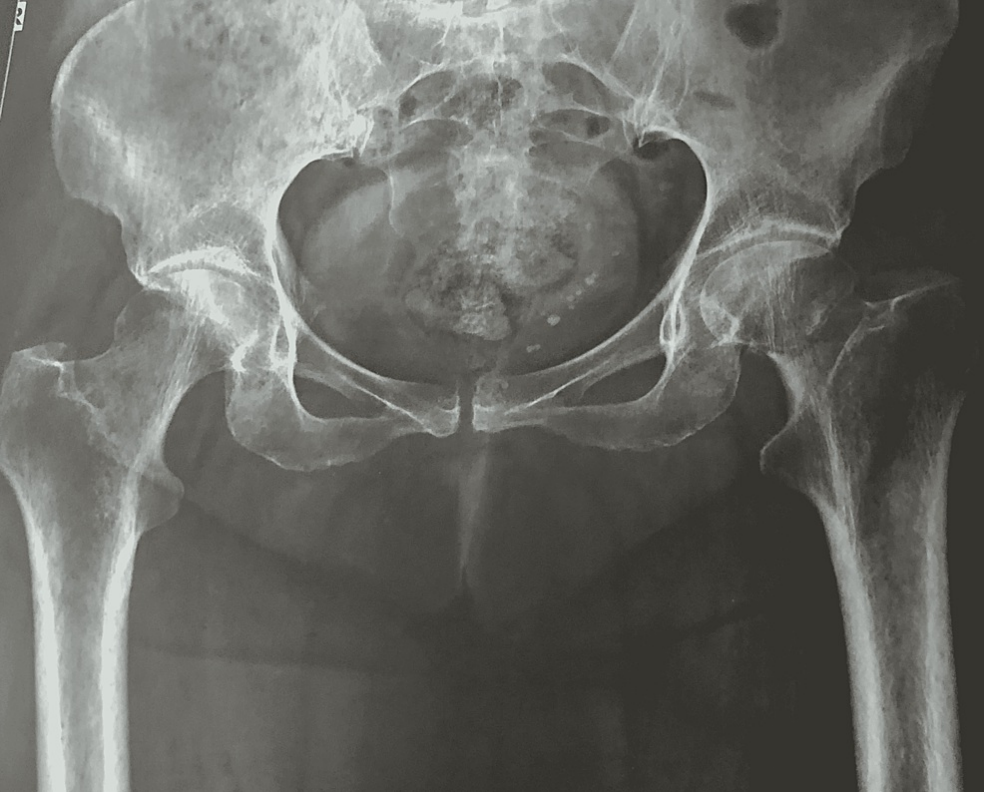
AP pelvis x-ray reveals a femoral neck fracture of the left hip

**Figure 2:**
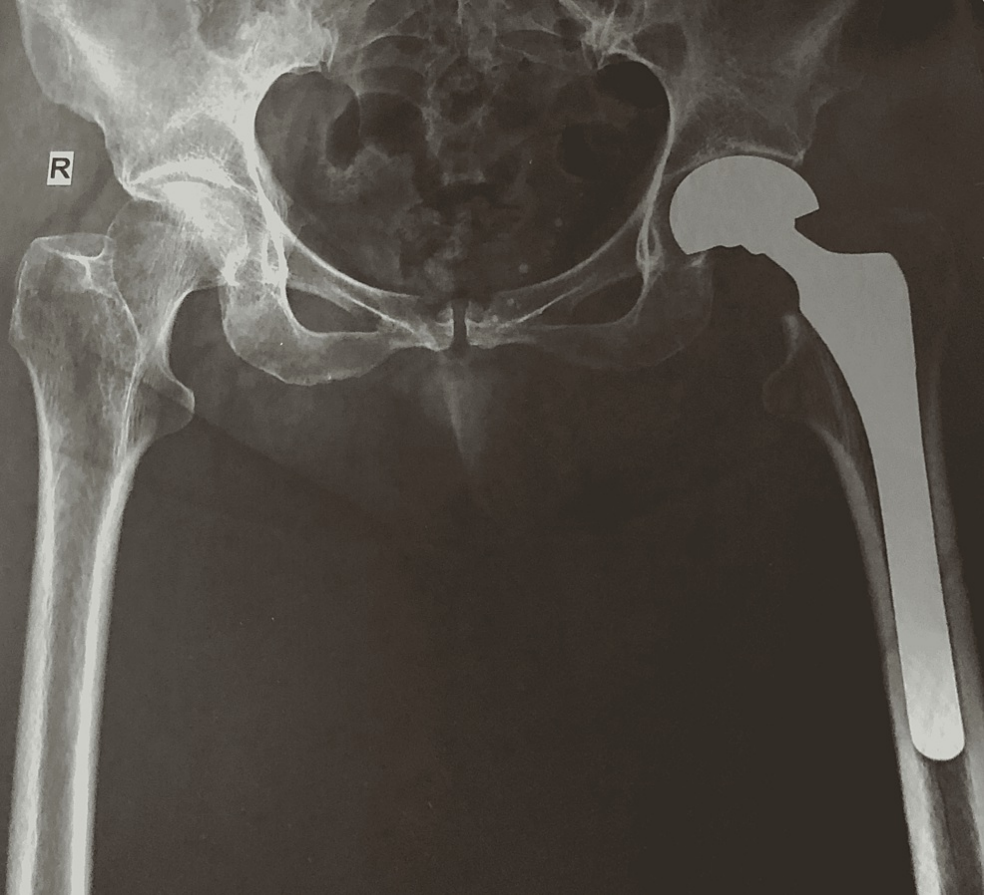
Postoperatively AP x-ray of the pelvis shows left hip hemiarthroplasty

A 64-year-old woman presented to our Accident and Emergency (A&E) Department complaining about tenderness, and swelling, of her left limb. The patient had sustained a femoral neck fracture of her left hip (Figure 1), which was addressed with a bipolar hemiarthroplasty twelve days ago (Figure 2). She otherwise had an unremarkable medical history and the perioperative and postoperative treatment was uncomplicated. On the fifth day postoperatively, she was discharged with normal radiological and laboratory examinations.

**Figure 3:**
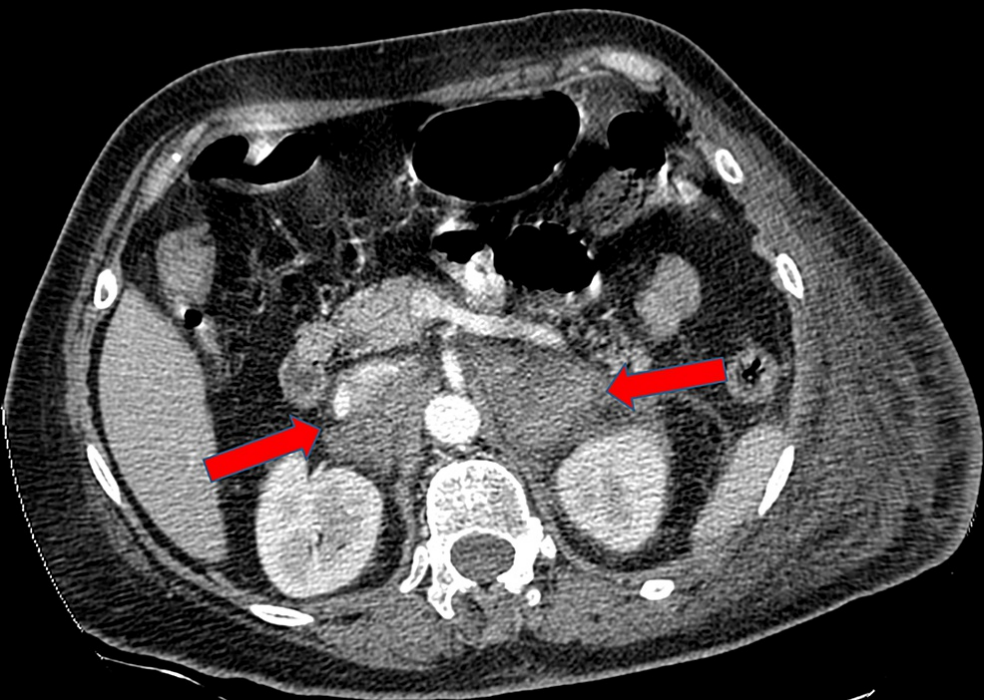
CT scan confirms bilateral adrenal hemorrhage. Adrenal congestion with adrenal thickening and periadrenal stranding. The adrenal hematoma appears oval with surrounding stranding of the periadrenal fat.

At the A&E Department, D-dimers were extremely increased (>1,000μg/L) and the duplex ultrasound revealed extended deep vein thrombosis of the common iliac vein and the deep femoral vein. The patient was prescribed Tinzaparin, 14.000 units once per day. The following day, she suffered from tachypnea, increased heart rate (110/min), shivering, and a fever of 38.2ºC. Her blood pressure was 85/55 mmHg (persistent hypotension despite fluid administration) and laboratory marks revealed serum sodium 125mmol/L (normal 134-145mmol/L), potassium 7.3mmol/L (normal 3.5-5.4mmol/L), creatine 1.25mmol/L (normal 0.8-1.2mmol/L), urea 22mg/dL (normal 6-24mg/dL), CRP 0.4 (normal <0.8), white blood cells 7,200 (normal 4,000-10,000), platelets 70.000 (normal 150,000-450,000), hemoglobin 8.2g/dl (normal 12.0-16.0g/dL). The assessment of cardiologists and pulmonologists indicated a differential diagnosis of pulmonary embolism, and a computed tomographic pulmonary angiography (CTPA) was performed. The CTPA inadvertently revealed BAH (Figure 3). The diagnosis of acute adrenal failure was confirmed by a short synacthen test [7]. The patient received 250mg of hydrocortisone immediately and her clinical situation was stabilized with normalization of blood pressure and temperature. The next day laboratory investigations revealed serum sodium 137mmol/L, potassium 4.32mmol/L, and platelets 68,000. Tinzaparin was substituted with fondaparinux sodium 7.5mg due to platelet count level. The fondaparinux dose remained therapeutic because of the extensive deep vein thrombosis and the high risk of pulmonary embolism. In the following days, the dose of hydrocortisone gradually decreased and a therapeutic protocol of prednisolone 5mg was prescribed for life due to installed adrenal insufficiency.

## Discussion

BAH is a rare postoperative complication after hip hemiarthroplasty and anticoagulation therapy preventing DVT. BAH usually induces adrenal insufficiency which causes severe morbidity and has high mortality rates [8,9]. Misdiagnosis is usual due to atypical clinical symptoms, so high clinical suspicion is necessary. The clinical situation includes abdominal pain, persistent hypotension despite fluid administration, hyponatremia, hyperkalemia, leukocytosis, and decreased hemoglobin [1].

Risk factors for non-traumatic BAH comprise thrombocytopenia, anticoagulation with low-molecule heparin as well as dabigatran, apixaban, warfarin, rivaroxaban, sepsis, major postoperative stress, coagulopathies- antiphospholipid syndrome, and HIT [3,10-14]. The gold standard for diagnosis is CT scan following clinical suspicion [15]. In our case diagnosis was done as a random finding while we were searching for pulmonary embolism.

The pathogenetic mechanism of BAH cannot be described precisely. We hypothesize that a subclinical HIT due to DVT and thrombocytopenia, and the stress after a major surgical operation prompted BAH. The patient’s clinical situation improved and stabilized immediately when hydrocortisone was administered. A hydrocortisone dose of 15-20mg is recommended daily due to established adrenal insufficiency [7].

## Conclusions

In conclusion, BAH and adrenal insufficiency are rare complications with usual misdiagnosis and high mortality rates. High clinical suspicion is crucial when patients undergo major surgical stress and a therapeutic anticoagulation regimen is prescribed. CT scan is the gold standard for diagnosis and the immediate administration of cortisone determines the clinical outcome and survival rate of these patients.
